# A System of Obstetrics by American Authors

**Published:** 1889-09

**Authors:** 


**A System of Obstetrics by American Authors.** Edited by BARTON COOKE HIRST, M. D., Associate Professor of Obstetrics in the University of Pennsylvania. Volume II. Illustrated with 221 engravings on wood. Philadelphia: Lea Brothers & Co. 1889.

The second volume of this valuable work aims to supplement the contributions to obstetric literature, published in the first volume, by embracing a wider range of subjects from the pens of American writers. A composite treatise in this department of medicine is a new venture, and possesses the merit, or advantage, of grouping a series of monographs from the ablest writers and teachers, which will command from the profession especial favor. This series of papers have been well selected, and the authors are men of marked ability in the special subjects assigned them. The editor has exercised rare judgment in the important position for which the publishers designated him, and the two volumes which have been published are works of real worth. In closely examining them, their purpose to aid the experienced obstetrician, rather than the student, is apparent. Even at the risk of the conflicting opinions, here and there given, there is a freshness and vigor in these monographs which increase their value and command the attention of the reader. We regard this feature one of great merit, and adds peculiar value to the work.

The first paper is from the pen of Dr. Theophilus Parvin, and embraces a wide range of interesting subjects, well grouped together, including Lacerations of the Uterus; Injuries of the Vagina, Vulva, and Perineum; Inversion of the Uterus; Hemorrhage during and after Labor; Eclampsia; Injuries to the Child during Birth, including the Plastic Changes occurring on the Head; Wounds of the Scalp and Face; Fracture of the Cranial Bones; Paralysis of the Facial Nerve; Injuries of the Muscles of the Neck; Injuries of the Arms, Trunk, and Lower Extremities. A valuable paper is given on Sudden Death during and following Labor, and on Diseases of the Mother, with reference to Labor. Dr. Parvin shows his long training and experience as a teacher, and his great experience as an obstetrician, in this contribution.

The Forceps and Embryotomy, by Dr. Edward P. Davis, of Philadelphia. The writer traces the history of the forceps back to the father of medicine, and gives due credit to the Chamberlens for reviving their use. The general indications for the use of the forceps are judiciously given. The axis-traction forceps, introduced in 1877 by

Tarnier, are not as forcibly urged on the general practitioner as we think their importance and value demand. While it may be true, as the writer states, "that the high application may not be necessitated for years in the practice of many physicians," the ordinary long forceps cannot fulfil the indications met by the axis-traction forceps. We regard Tarnier's instrument to be a most valuable addition to the armamentarium of the obstetrician, and, in our opinion, it should supplant all others in the high operations.

The subject of Embryotomy is ably discussed by the same writer, and also the indications and methods for its performance, and the instruments used. The question of choice between embryotomy or craniotomy, and the modern Cesarean section, gastro-elytrotomy, Porro's, or the Porro-Müller, operation, is discussed from the basis offered by statistics, rather than from the high moral principles involved. On this point, the author is open to criticism. The advances made in abdominal surgery makes the future of the Cesarean section as an elective procedure, in cases in which the fetus cannot be delivered *per vias naturales*, full of promise. The trend of professional thought is in favor of the improved Cesarean section. Our American operators, even if "they do not equal those of Germany, who are far in advance of all others," are making rapid strides for supremacy, which we confidently believe they will reach.

The Premature Induction of Labor is from the pen of Dr. James C. Cameron, of Montreal, and is an important contribution to the literature of the subject. A paper on Version is from the same author.

The Cesarean Operation, Symphysiotomy, Laparo-Elytrotomy, and Laparo-Cystectomy, by Robert P. Harris, M. D., of Philadelphia. The histories of these major operations in obstetrics are given, also the indications, the technique, and the new methods, and the results. The author gives a fair statement of the value of each method, without making any comparison of their advantages, and his paper is all the more valuable on this account. The reader is supposed to be able to draw his own conclusions.

Dr. Henry J. Garrigues, of New York, the apostle of antisepsis in midwifery in this country, presents a monograph on Puerperal Infection. The microbic theory in the causation of this scourge of the lying-in-room is fully endorsed. He believes the domain of the microbe is widening more and more, and leads us to infer, also, that the benign forms of fever and inflammation connected with the puerperal period arise from this cause. The term puerperal fever is discarded by the writer, and septicemia is not favorably considered. Puerperal infection suits the author much better, and is adopted, as a term which points out where the disease comes from, and teaches the proper measures to prevent it. Dr. Garrigues wisely limits the discussion of the nature of puerperal infection, its etiology, pathology, symptoms, diagnosis, and prognosis, and devotes much space to a detailed description of the means by which infection is prevented and treated. Herein is the value of this paper to the practical obstetrician, and we find it full of the most minute directions, all of which are valuable, and, if strictly carried out, afford a rational prophylaxis and an effective treatment for this disease.

Dr. Garrigues also furnishes a paper on Inflammation of the Breasts and Allied Diseases connected with Child-birth, including sore nipples, deep inflammation of the nipples, eczema of the areola, cellulitis and adenitis of the areola, erysipelas of the breasts, lymphangitis of the breasts, mastitis, cold or chronic inflammation of the breasts, swelling and milk-retention in the axilla, mastitis of the new-born, and fistula of the breasts.

Etiology of Puerperal Fever, by Harold C. Ernst, M. D., of Boston. The writer aims to show the nature of the so-called puerperal fever as it appears in the light of modern research. First, That puerperal fever is not a specific disease. Second, That these infectious wound-diseases are never endogenous, but always exogenous, in origin. Third, They are produced, in all cases, by the action of living ferments.

Some Complications of the Puerperal State, independent of Septic Infection, by Barton Cooke Hirst, M. D., Philadelphia. The title of this section, Some Complications, gives the writer a wide scope, and we find here a grouping of non-septic conditions and diseases which, if fully considered, would make up a complete volume. The necessity for condensation, in order to treat the great variety of subjects, renders fullness and clearness of description impossible. The range of subjects is great, viz.: involution and its abnormalities, displacement of the uterus, dislodgment and disintegration of clots at the placental site, puerperal hematoma, non-infectious fevers, constipation, pneumonia, pleurisy, the exanthemata, puerperal diphtheria and malaria, rheumatism, gonorrhea, diseases of the urinary system, abnormalities of the milk secretion, relaxation of the pelvic joints, etc. The author evidently intended to omit but few of the complications of the puerperium, and in this respect at least he has gathered much valuable matter, which will aid the obstetrician in his researches.

Insanity and Diseases of the Nervous System in the Child-bearing Woman, by James Hendrie Lloyd, M. D., of Philadelphia. Dr. Lloyd divides the subject into two general divisions: First, Puerperal

Insanity and the Insanity of Pregnancy and Lactation, which includes the definition of puerperal mania, its history, frequency, causes, symptoms, diagnosis, prognosis, pathology, and morbid anatomy, and treatment; the influence of pregnancy, parturition, and lactation on insanity; the medico-legal aspects of the insanities of pregnancy, the puerperium, and lactation. Second, Occasional Neuroses of Pregnancy and the Puerperium, including cerebral embolism and hemorrhage, chorea of pregnancy, tetanus following abortion and labor, tetany, epilepsy in pregnancy, cerebral hemorrhage and embolus in pregnancy and the puerperium, and pressure palsies following labor. The writer presents a most valuable paper, which we have examined with great interest and with profit.

The Management and Diseases of New-born Infants, by J. Lewis Smith, M. D., of New York. The reputation of Dr. Smith is a guarantee of the value of this monograph. He treats of the care of the infant, congenital malformations, as acrania, meningocele, encephalocele, hydrencephalocele, spina-bifida, congenital abnormalities of the circulatory system, tetanus or trismus neonatorum, ophthalmia neonatorum, (purulent and gonorrheal), icterus, umbilical regulations, and hemorrhage, sepsis of the new-born, diphtheria, thrush, hematemesis, and melena neonatorum, diarrhea, constipation, etc. The subjects are briefly considered, and the condensation aimed at by the author makes the paper all the more valuable to the general practitioner, who cannot examine more voluminous works.

This review may be summarized by stating that, in the selection of writers and subjects, the editor and publishers have shown commendable judgment, and, as a result, have presented to the profession a work of rare value, which we are confident will be well received and appreciated.

